# A study protocol for mixed-methods evaluation of the structure, design, and availability of medical student wellbeing programs

**DOI:** 10.1371/journal.pone.0351759

**Published:** 2026-06-18

**Authors:** Neel Godbole, Sugy Choi, Kwanbo Shim, Yuning Liu, Jason Hu, Jennifer Wong, PhiYen Nguyen, Sze Wan Celine Chan, Lan Doan, Nelson Lin, Vanessa Salcedo, Stella S. Yi

**Affiliations:** Department of Population Health, New York University Grossman School of Medicine, New York, New York, United States of America; University of Huelva: Universidad de Huelva, SPAIN

## Abstract

**Introduction:**

In recent years, there has been growing concern over the wellbeing and mental health of medical students in the United States, driven by the academic, personal, and professional challenges inherent in medical school. Recent data indicates that medical students experience higher rates of psychological stress, anxiety, and depression compared to the general population, with the COVID-19 pandemic exacerbating these challenges. Medical student suicide, linked to burnout and depression, highlights the urgent need for effective wellbeing support. Despite the documented barriers to mental wellbeing, such as self-imposed pressures, imposter syndrome, stigma around help-seeking, and financial difficulties, medical student wellbeing programs remain understudied at the structural and design level.

**Methods:**

This is a multi‑phase qualitative study (sequential-exploratory) that combines a web-based environmental scan and content analysis with key informant interviews and focus groups, using methodological triangulation to develop a framework for evaluating wellbeing programs. First, we will conduct a web-based content analysis of publicly available resources across medical school websites. We will identify key characteristics of wellbeing programs, such as mental health resources, structural well-being components, and culturally integrated approaches. Then, we will conduct key informant interviews with medical school administrative staff to discuss wellbeing programs in detail and hold focus group interviews with medical students to gather their perspectives on how to improve their health and wellbeing. Based on the findings from these three components, we will develop a comprehensive and standardized framework for evaluating medical school wellbeing programs that can be used across institutions.

**Ethics and dissemination:**

Human Research Ethics Approval was obtained from the NYU Langone Health Institutional Review Board (IRB ID: i25-00965). The content analysis results and qualitative themes extracted from key informant and focus group interviews will be made available to all study participants. They will also be disseminated in a peer-reviewed journal.

## Introduction

Medical students experience significant burnout [1] and higher rates of anxiety compared to the general population [2], along with disproportionately high prevalence of self-reported depression and suicidal ideation [3]. The emotional health of medical students has been found to decrease over their training, with students experiencing the greatest deterioration in their health during the first year [4]. Medical student wellbeing is a broad phrase describing these students’ present physical, emotional, and mental health [4]. Decreased wellbeing among medical students can lead to poor academic performance, heightened cynicism, academic dishonesty, substance abuse, and suicidal ideation, and may contribute to depression, stress, anxiety, and burnout [5]. Structural features of medical education, such as competitive evaluation systems, limited institutional support, and pervasive stigma around mental health, further compound these challenges [6]. These concerning factors highlight a troubling paradox in medical education: the very students who will one day care for the health of others are themselves grappling with increasing psychological distress.

While most medical schools in the United States provide their students access to mental health services [7], this has not been enough to overcome barriers to holistic wellbeing that medical students face. Firstly, students’ highly conscientious approach towards their academics has been implicated in students’ experiences of psychological distress [8,9]. Further, the environment in today’s medical education system is often described as a ‘culture of perfection,’ making it harder for students to admit vulnerability and leading to heightened stress and burnout [10]. Overwhelming academic, professional, and personal stressors result in a lack of time that prevents medical students from seeking out wellbeing resources [11]. Students additionally cite financial difficulties, lack of awareness about resources, and fear that help-seeking could impact their professional outcomes [11]. Stigma also presents a potent barrier to student wellbeing. It was found that first- and second-year preclinical students were more likely than their clinical-year peers to report that depressed students had lower levels of intelligence, presented a danger to their patients, and were incapable of coping with the stressors of medical education [12]. These stigmas, along with the popular, yet misleading assumption that physicians should be invincible [13], may underlie student hesitation around seeking outside resources.

The American Association of Medical Colleges (AAMC) Committee on Student Affairs (COSA) Working Group on Medical Student Wellbeing, which primarily consists of administrative wellbeing or student affairs deans from across the country, has developed educational resources, including systemic recommendations, programming strategies, wellbeing curricula, and evaluation tools [14]. This group has underscored the need for systematic approaches and research to assess medical student wellbeing [14]. Further, the American Medical Association (AMA) has a selection of wellbeing-focused articles for diverse audiences, including students and educators alike. As of May 2025, the accrediting body for medical schools across the United States, The Liaison Committee on Medical Education (LCME), requires schools to offer “programs to promote [students’] wellbeing and to facilitate their adjustment to the physical and emotional demands of medical education” [15,16].

Previous work has proposed a framework for grouping these wellbeing programs into four distinct categories: preventative, reactive, structural, and cultural [16]. Preventative programming includes forward-thinking interventions that preemptively tackle stressors in medical education. Reactive programming is designed to offer resources to students who are actively experiencing distress. Structural programming can assess and act on an institution’s overall learning environment and curriculum. Finally, cultural programming includes institution-level practices such as leadership modeling healthy behaviors or providing administrative support, aligning programs with student wellbeing priorities, and systematically collecting and responding to student feedback on wellbeing initiatives [16].

To date, however, there is still limited literature detailing how these program categories take shape at different medical schools across the United States. To the study team’s knowledge, there are only two peer-reviewed studies addressing this question [16,17]. The first study surveyed 32 medical schools, and it investigated the extent to which schools offered wellbeing initiatives across the emotional/spiritual, physical, financial, and social domains [17]. A later survey study of United States and Canada medical schools found that 93% of respondents had a formal wellbeing program available for students, and it provided information about the scope of wellbeing programming across these four distinct domains [16]. While most medical schools reported sufficient preventative and mental health programming, there was a notable lack of structural changes, such as pass/fail clerkships or the elimination of competitive honors, that could address curricular drivers of student distress [16]. The study emphasizes the need for more research on structural interventions, alignment between wellbeing messaging and institutional policies, and adequate cultural and administrative support to create truly effective and sustainable wellbeing programs [16].

Unlike prior survey studies that focus on describing the presence and content of wellbeing programs at LCME-accredited medical schools, the present study uses a multiphase mixed method approach, including Natural Language Processing (NLP) assisted web-scraping of publicly available data, qualitative interviews with school administration, and focus groups with medical students, to provide a more comprehensive understanding of wellbeing programming. Web-scraping is a tool that can capture publicly available, school-specific wellbeing information relevant to medical students. Unlike previous studies that capture only a small proportion of medical schools that were willing to respond [16,17], our web-scraping approach captures a random sample of institutions, ensuring a more representative dataset. Additionally, this study uniquely aims to develop a standardized evaluation framework for wellbeing programs, something previous work has not offered. While the prior two studies in this space identified valuable gaps in structural interventions and the importance of cultural and administrative support, the proposed study directly engages a broader, non-selective sample of stakeholders to identify actionable strategies and evaluate effectiveness. The overarching goal is to inform evidence-based improvements across institutions. This protocol represents original work and does not duplicate previously published studies; the proposed analyses and results have not been disseminated elsewhere.

## Materials and methods

### Study design

**Fig 1:**
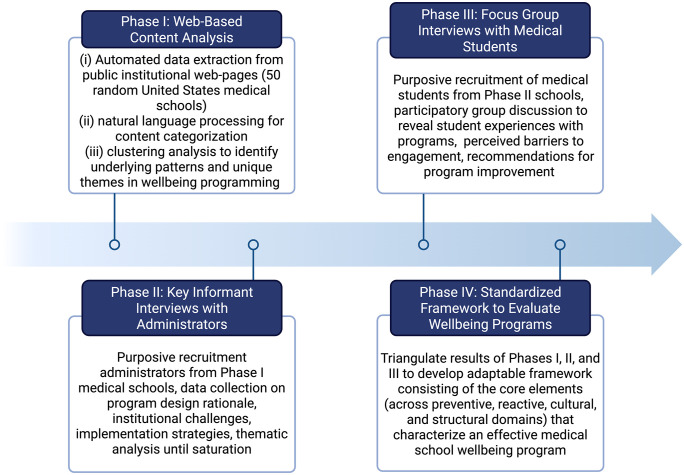
Descriptions and timeline of phases I, II, III, and IV.

This study aims to employ a sequential multiphase mixed methodology, consistent with a sequential exploratory approach, in which a computational analysis of institutional websites informs subsequent qualitative data collection through administrator interviews and student focus groups to evaluate medical student wellbeing programs in the United States (Fig 1): (1) computational analysis of institutional websites, (2) semi-structured interviews with administrators, and (3) student focus groups. The computational component provides structured, quantitatively informed insights into institutional content, while the qualitative phases explore contextual meaning and lived experiences. This study aligns with the sequential exploratory framework outlined in [18] and is similar to the overall research design in [19,20], in which close-ended data was first collected quantitatively followed by open-ended data collection from qualitative components. However, unlike [19,20], priority will be given to the qualitative components of this study, with the preliminary computational phase serving an exploratory and instrument-development function that informs the subsequent phases. Phase I leverages a web-based content analysis of wellbeing programs using NLP techniques. Consistent with explanatory connecting procedures in mixed-methods research, findings from the computational phase will directly inform subsequent qualitative sampling and instrument development. For example, institutional characteristics identified during website analysis (e.g., variation in visibility, comprehensiveness, or framing of wellbeing resources) will be used to guide purposive recruitment strategies and refinement of interview and focus group guides. Phases II and III both entail use of participatory research methodologies, which, by “[engaging] community stakeholders in the research process” through group or individual conversation, can lead to the “production of data that are more adequate and relevant for [the stakeholders themselves]” [21]. Insights from administrator interviews subsequently shape the focus group discussions with students. The objective of Phase IV is to synthesize the findings across phases. We plan to triangulate information sequentially to generate a comprehensive understanding of the study objectives. We plan to develop a standardized, adaptable framework for evaluating institutional wellbeing programs, with the end goal of improving administrative program design and medical student wellbeing in the US. Integration will occur iteratively across phases through comparative matrices, triangulation procedures, and joint displays that link computational findings with qualitative themes to examine areas of convergence, complementarity, and discordance across institutional messaging and stakeholder experiences.

The research team includes investigators trained in qualitative and mixed-methods research with a focus on health equity and structural determinants of health. Our collective experiences in clinical care, policy, and community-based research inform our interpretive lens, including attention to power, context, and lived experience. We recognize that these perspectives may shape data interpretation and will engage in ongoing reflexivity through memoing, iterative coding, and team-based discussions. Given the protocol nature of this study, team composition may evolve over time.

### Study population

The study population comprises institutional wellbeing program administrators and undergraduate medical students who have participated in wellbeing programs at U.S. Doctor of Medicine (M.D.) granting medical schools (Phases II and III). These institutions are all accredited by the same governing body, the LCME, and therefore are expected to share overlapping institutional priorities and educational structures. To minimize heterogeneity arising from variation in institutional accreditation and training level, Doctor of Osteopathic Medicine (D.O.) granting medical institutions, international medical schools, and medical trainees beyond the undergraduate medical education level (e.g., residents and fellows) will be excluded. Table 1 summarizes the type and characteristics of participants, sample size, and how the sample will be recruited for Phases II and III.

**Table 1:** Phase II and III sample characteristics.

Participant Groups	Participant Types and Description	Sample Selection and Strategy	Anticipated Sample Size
Medical School Administrators	Dean of Student Affairs, Director of Student Wellbeing, Director of Student Support Services	Purposive sampling to identify individuals with significant oversight of student wellbeing	At least 25 KIIs^b^ with administrators (one per institution)
	An individual who directly contributes to the design of their institution’s undergraduate medical student wellbeing program	Participants chosen from Phase I institutions; participants will vary by institutional size, geographic region, and resource level	
Medical Students	Preclinical, clerkship, or post-clerkship medical student actively enrolled in an M.D.^a^ program	Purposive sampling to identify interested students through institutional listservs, social media, and student wellbeing offices	Three FGDs^c^ of 5–8 participants each, for a total of 15–24 participants
	An individual who has engaged with their institution’s wellbeing program at any point over the course of their medical education	Screening surveys will ensure diversity by educational stage, gender, racial/ethnic background, and level of engagement with wellbeing programs; students at similar stages of training assigned to the same FGD^c^	

Note: ^a^ Doctor of Medicine (M.D.).

^b^Key Informant Interview (KII).

^c^Focus Group Discussion (FGD).

### Study phases

#### Phase I: Webpage-based content analysis using natural language processing (NLP) techniques.

Wellbeing program information publicly available on medical school webpages will be collected and analyzed using a combination of automated data extraction, data categorization, NLP, and clustering techniques. A random sample of 50 medical schools will be selected from a list of fully accredited MD-granting medical schools from the LCME website. Random selection will be implemented using a reproducible procedure with a fixed random seed to ensure transparency and replicability. If a school has insufficient publicly available information about its wellbeing programming, no further effort will be made to collect this data, and the school will be replaced by an alternative randomly selected institution from the original list. Information is considered insufficient if only general statements about wellbeing are made without describing at least one wellbeing program or service for medical students. To extract relevant data from each school’s website, we will use a custom Python-based crawling pipeline built with open-source libraries such as requests and BeautifulSoup, to discover, filter, download, clean, and segment relevant pages from each school’s website (Table 2). It is optimized to accommodate the varied web structures, dynamic content, and nested documents across different institutions’ websites.

**Table 2:** Technical summary of Phase I.

Component	Purpose	Approach
School sampling	Define the study sample	• Randomly select 50 fully accredited LCME MD-granting medical schools
Data collection	Gather publicly available wellbeing information	• Use Python-based web extraction pipelines to identify, retrieve, clean, and segment relevant website content across institutions
NLP-based relevance filtering	Identify the most relevant text for analysis	• Tokenize text into sentences and use embedding-based similarity scoring to rank Retain sentences most responsive to predefined questions
Question answering	Answer predefined questions about wellbeing programs	• Use a fine-tuned BERT model to identify question-relevant keywords Extract the most likely answer and confidence scores will be recorded.
Embedding and dimensionality reduction	Prepare text for pattern detection and visualization	• Convert text into numerical embeddings and reduce dimensionality with UMAP to a 2- or 3-dimensional representation
Clustering and labeling	Identify semantically related groups of wellbeing content	• Apply HDBSCAN to group related texts and isolate outliers • Apply c-TF-IDF with Maximal Marginal Relevance to generate descriptive, diverse, and non-redundant cluster keywords.

* The code used in this study will be made publicly available at https://github.com/JosephKBS/medschool-wellbeing upon publication.

The extracted content will be categorized using regular expression patterns applied to website URLs and texts into one of four programmatic domains: preventative, reactive, cultural, and structural. If no match is detected, the content will be classified as “General Information.” Relevant sentences within each category will be identified using embedding-based similarity analysis. Text will be tokenized into individual sentences and ranked by cosine similarity to predefined question embeddings using a sentence-transformer model. Sentences below a similar threshold will be filtered out. Gaps in services or inconsistencies in how wellbeing programs are represented will also be noted. Metadata, such as the publication date of information and webpage URLs, will be recorded for context. Through external python packages that assist in data processing, scientific calculation, machine learning, and natural language processing (e.g., numpy, pandas, nltk, sklearn, and torch packages), a list of keywords from each of the four program categories will be used to locate relevant sections of each website. At this stage, the custom-built tool will extract more structured data, including page titles, subheadings, program descriptions, and hyperlinks to wellbeing resources.

Once full text data has been collected from each institution, a set of predefined questions about wellbeing programs will be answered using a fine-tuned BERT (Bidirectional Encoder Representations from Transformers) model. The model will extract keywords from each question and check for their presence in the text. If no relevant keywords are found, the model will return “No keywords found in context.” If keywords are present, the model will identify the most likely answer tokens. The answers generated will be cleaned and formatted for clarity, with references and confidence scores recorded.

To address limitations of direct language learning model (LLM) based responses, such as missing content, infrequent updates, or inaccessible webpages, we will apply clustering methods to identify underlying patterns. Text data will be embedded into numerical vectors, and dimensionality will be reduced using Uniform Manifold Approximation and Projection (UMAP). This step simplifies the complex numerical data into a 2 or 3-dimensional map, making it easier to visualize and process without losing the importance of structural relationships among data points. The planned embedding model is “all-miniLM-L6-v2”, and consine similarity will be used for semantic matching. Dimensionality reduction will be performed using Uniform Manifold Approximation and Projection (UMAP) with the following planned parameters: n_neighbors = 15, min_dist = 0.1, n_components = 2, and random_state = 42.

We will then apply the Hierarchical Density-Based Spatial Clustering of Applications with Noise (HDBSCAN) algorithm to group semantically and contextually related texts. Unlike standard grouping methods that push every data point into a circle, this algorithm is flexible enough to find irregularly shaped groups and ignore outliers that do not fit anywhere. Cluster keywords will be extracted using class-based TF-IDF (c-TF-IDF) with Maximal Marginal Relevance (MMR) to ensure keyword diversity. Clustering will be performed with min_*cluster*_size = 5, and min_samples = 3. Collectively used, these two methods will help label each group with words that are not only descriptive but also distinct from one another, preventing semantically repetitive or redundant tags that do not meaningfully differentiate between clusters (e.g., ‘exercise’, ‘physical activity’, and ‘fitness’). The final analysis will include a de-identified list of participating schools to ensure confidentiality while allowing for site-level analysis and interpretation of contextual factors. For quality assurance, we will perform qualitative review of text samples within each cluster and examine the parameter sensitivity to confirm the robustness of the cluster structures.

To further enhance reproducibility, analytic scripts and supporting documentation will be made available to the extent permitted by institutional and platform constraints. Final model versions, packages, and parameter files will be archived in the repository to support reproducibility.

#### Phase II: Key informant interviews with medical school administrators.

Medical school administrators are selected as the key informants for this study because of their community-facing and leadership roles at their institutions, and the higher-level information they offer about wellbeing programs that students cannot [22]. They represent an intrinsically bound group that will provide detailed information about program policies, institutional priorities, and structural barriers to program design or student engagement [22].

Given the small and well-defined nature of the medical school administrator sample, a purposive, non-probabilistic sampling strategy will be used to recruit at least 25 individuals (one per institution) with primary oversight of student wellbeing from LCME-accredited medical schools during Phase I of this study (see Table 1). Administrators will be selected according to the selection criteria outlined in Table 3. For institutions in which these administrators are less publicly visible or more difficult to connect with, snowball sampling will be used to reach the administrator [22]. All prospective participants will be contacted by email with IRB-approved recruitment materials, including digital flyers, study information, and a REDCap electronic consent form. In this key informant sampling approach, issues of data sufficiency may arise from a lack of administrator availability due to ongoing professional and organizational demands that may take precedence over study participation [22], or from non-response to email communications. If a single administrator cannot be contacted or identified as a key informant at a given institution, another institution will be chosen. Three attempts will be made to contact administrators at the current institution before considering an alternative institution.

**Table 3:** Phase II participant selection criteria.

Category	Criteria
Inclusion Criteria	1. At least 21 years old. 2. Identifies as a medical school administrator who has key responsibilities related to wellbeing program development or administration (e.g., Dean of Student Affairs, Director of Wellbeing, among others) and is willing to provide detailed information about institutional priorities 3. Has experience with their institution’s wellbeing program, whether through program design or supporting individual or groups of students 4. Employed at an LCME^a^-accredited U.S. MD^b^-granting medical institution
Exclusion Criteria	1. Not willing to give consent for participation 2. Has a role not directly relevant to student wellbeing program (e.g., informal mentors without formal program responsibilities or counselors/psychiatrists at institution’s counseling center)

^a^Liaison Committee on Medical Education (LCME).

^b^Doctor of Medicine (M.D.).

Following electronic consent, 1-hour virtual, semi-structured, and open-ended key informant interviews (KIIs) will be conducted with participating administrators to explore institutional approaches to developing, implementing, and sustaining student wellbeing programs. Before the start of each interview, verbal consent will be reaffirmed, and interviews will be audio/video-recorded and transcribed verbatim. The content of the discussion will vary by interviewee and institution, but several overarching domains, each associated with a set of broad guiding IRB-approved questions, will be gathered from each conversation: the administrator’s background and role, wellbeing program content, program design processes, mechanisms for long-term sustainability and support, methods of evaluation and improvement, collaborations and best practices, institutional prioritization of wellbeing, and future program directions (Table 4). These questions will be sufficiently broad to avoid constraining the administrator’s responses, encouraging them to freely respond and draw on their full expertise and experience [22].

**Table 4:** Key informant interview guiding themes.

Guiding Theme	Discussion Questions
Administrator Background and Role	Can you describe your role and involvement with the medical school’s wellbeing program?
	How long has the wellbeing program been in place at your institution?
Program Content	What key components or topics does your program cover?
	How do you decide what content to include?
	Are there particular wellbeing challenges among students that your program targets?
Design Process	Can you describe the process used to initially develop the wellbeing program?
	Who was involved in designing and approving the program?
	How do you incorporate feedback from students or staff?
Sustainability and Support	How do you measure the effectiveness of your wellbeing program?
	Have you made any changes based on internal evaluation or new evidence?
Collaboration and Best Practices	Do you collaborate with other departments or external organizations in your wellbeing initiatives?
	Are there best practices or conceptual models for wellbeing programming that you follow or recommend for other schools?
Institutional Prioritization of Wellbeing	Relative to other priorities, how important is wellbeing to your institution?
	What are the challenges for making wellbeing more of a priority?
Future Directions	What plans or ideas do you have for evolving or expanding your wellbeing program?
	What potential changes to programs would be most impactful and realistically feasible?

Two trained researchers will independently code a subset (N = 5) of transcripts in a double-blind manner using the Dedoose software. Data analysis will employ a thematic analysis approach using a hybrid inductive-deductive coding strategy, in which codes are developed inductively from the interview data and informed by preliminary findings from the web-scraping content analysis. An iterative approach will be employed to refine the codebook, and each newly identified theme will be represented by a unique code. This process will identify recurring themes related to program design rationale, institutional priorities, implementation challenges, and strategies for sustainability. Inter-coder reliability will be assessed using Cohen’s kappa after the first five interviews. Substantial discrepancies in coding, indicated by kappa values ≤ 0.50, will prompt group discussion, clarification of coding strategies, and consensus-based resolution [23].

Data saturation will be evaluated throughout the coding process in accordance with the previously proposed framework for operationalizing saturation [24]. New codes or distinct themes that those interview transcripts yield will be updated in the codebook. Further, any modifications to code definitions resulting from team discussion will also be documented iteratively. Data saturation will be considered achieved when a substantial decline in the number of newly identified themes is observed in subsequent phases of analysis [24].

#### Phase III: Focus group discussions with medical students.

Focus group discussions (FGDs) place special emphasis on understanding the interactions and dynamics among research participants [25]. We will conduct approximately three 1.5-hour-long virtual and semi-structured FGDs, each with 5–8 participants for a total of 15–24 student participants. Non-probabilistic, purposive sampling will be used to recruit participants from the same set of institutions studied in Phase II to ensure alignment between administrative and student perspectives.

Recruitment will leverage the existing contacts with wellbeing offices and key informants from Phase II, with whom the research team will work to disseminate IRB-approved recruitment materials to students via institutional channels such as listservs, social media accounts, and departmental communications. Table 5 outlines inclusion and exclusion criteria for medical students. Snowball recruitment will also be employed, but participants recruited via this method will not be placed in the same FGD as the individual who originally referred them to the study [25]. A REDCap survey will be used to screen prospective participants for having interacted with their institution’s wellbeing program at any point during their medical education and if they are willing to discuss personal experiences within a group setting.

**Table 5:** Phase III participant selection criteria.

Category	Criteria
Inclusion Criteria	1. At least 21 years old 2. Identifies as an undergraduate medical student^a^ at an LCME^b^-accredited U.S. M.D.^c^-granting medical institution 3. Has interacted with institution’s wellbeing program in the past and is willing to discuss their personal experiences and feedback about facets of the program
Exclusion Criteria	1. Not willing to give consent for participation 2. Has engaged with third-party wellbeing services (e.g., independent counseling/therapy) but not their institution-sponsored wellbeing program

^a^Preclinical, clerkship, or post-clerkship stage student (not a resident or fellow).

^b^Liaison Committee on Medical Education (LCME).

^c^Doctor of Medicine (M.D.).

After students are determined to be eligible participants, a member of the research team will contact them via email to schedule participation in a FGD. The email will offer a range of available dates and times, as well as include a secure scheduling link to facilitate coordination based on participant availability. Participants will be asked to confirm their selected session and will receive a calendar invitation with additional details, including a REDCap electronic consent form, virtual meeting link, and contact information for the study team. Group composition is also important to consider, as it can alter group dynamics or affect participants’ willingness to share opinions [26]. When assigning participants to FGDs, the research team will aim to strike a balance between homogeneity and heterogeneity. Groups will be kept homogenous along the most salient domain, stage of training (i.e., preclinical, clerkship, post-clerkship), to ensure shared context for FGD participants and reduce the likelihood of hierarchical dynamics influencing discussions. Simultaneously, the team will consider varying groups by sex, race or ethnic background, institution location, and extent of previous engagement with wellbeing activities.

FGDs will be moderated by a trained primary and secondary moderator [25,26]. Upon reaffirming verbal consent with all participants and reiterating the anonymity of their responses, each session will begin with a brief group activity that primes participants for an open conversation ahead, along with short introductions by group members. The primary moderator will facilitate the conversation based on several open-ended guiding questions and ensure equitable student participation by inviting contributions from participants who may speak less frequently in the presence of more dominant peers [25,26]. When group discussion significantly goes beyond the scope of the study’s aims, the primary moderator will be responsible for refocusing the conversation. The primary moderator will refrain from making any opinionated comments that may inadvertently influence group dynamics [25]. Meanwhile, the secondary moderator will take field notes to capture non-verbal cues, such as facial expressions, attitudes, and tone, all of which will be important to contextualize participant responses during qualitative analysis. All sessions will be audio/video recorded and transcribed verbatim.

The content of the FGD will center around several guiding themes with IRB-approved associated questions (Table 6), including broader experiences, program content, structure and delivery, accessibility and inclusivity, impact and outcomes, suggestions for program improvement, and opinions on program framework development. This discussion guide will be continuously updated by the research team based on participant discussion, since students may raise program issues or topics not included within the guide.

**Table 6:** Focus group discussion guiding themes.

Theme	Discussion Questions
Overall Experiences	Can you describe your overall experience with your medical school’s wellbeing program?
	What aspects of the wellbeing program have been most helpful to you?
Program Content	What topics or services do you think are essential in a medical school wellbeing program?
	Are there important issues that have been overlooked or under-addressed at your institution?
Program Structure and Delivery	How are wellbeing activities or resources typically offered? (e.g., workshops, counseling, peer groups)
	What formats or approaches to wellbeing do you find most engaging and accessible?
	How often do you think wellbeing activities should be available?
Accessibility and Inclusivity	Are wellbeing resources easy to access when needed?
	Does the program adequately support diverse student needs, including mental health, physical health, social support, and cultural aspects of student life?
	What barriers, if any, have you encountered in using your institution’s wellbeing services?
Impact and Outcomes	What impact do you think wellbeing programs have had on student success (broadly defined) and wellbeing overall?
Suggestions for Improvement	If you could design the ideal wellbeing program, what would it include?
	How could medical schools better support student wellbeing?
	What roles should faculty, staff, and students play in wellbeing initiatives?
Framework Development	What core elements should be included in a standardized medical school wellbeing program framework?
	How flexible should such a framework be to accommodate different schools or student populations?

Transcripts will undergo thematic analysis via Dedoose, evaluation of interrater reliability, and retrospective data saturation analysis through methods similar to those described in Phase II. Codes and emerging themes will be compared with those from KIIs to identify areas of convergence and divergence between administrative intent and student experience.

#### Phase IV: Creation of standardized framework to evaluate medical student wellbeing programs.

Integration of data will occur during design (connecting phases), analysis, and interpretation, specifically between Phases I and II and Phases II and III. Findings will be triangulated using structured approaches, including comparative thematic matrices and joint displays [27], to identify areas of convergence and divergence across data sources and to inform development of a standardized framework. For instance, if computational findings suggest a trend or similarity in the format of wellbeing offerings at medical schools that is also highlighted by administrator and student input, this may emerge as a convergent theme. The final framework will draw from all three components of the project: (1) the web-scraping content analysis of institutional wellbeing programs (Phase I), (2) KIIs medical school administrators (Phase II), and (3) FGDs with medical students (Phase III). Together, these complementary data sources will be triangulated to identify convergent, complementary, and divergent themes related to the design, implementation, and sustainability of medical student wellbeing programs. Specifically, the content analysis will provide a systematic overview of publicly articulated program structures, resources, and stated institutional priorities, while administrator interviews will offer contextual insight into program rationale, decision-making processes, and implementation constraints. Student interviews will contribute experiential perspectives on program accessibility and perceived effectiveness. Themes emerging across these three components will be synthesized to define a set of core elements that characterize effective and sustainable wellbeing programming.

The collected results will be mapped onto the evaluative framework proposed by our colleagues [16], consisting of preventative, cultural, reactive, and structural programming components. Following initial development, the draft framework will undergo review by an expert panel with diverse expertise in medical education, student wellbeing, and related fields. Panelists will assess the framework using structured prompts focused on content validity, clarity, and feasibility. Feedback will be collected using standardized forms, synthesized across reviewers, and incorporated through iterative revisions. Where needed, a structured consensus process will be used to resolve areas of disagreement and guide final framework refinement.

The finalized framework emerging from this study will serve as a practical tool for medical schools to comprehensively assess existing wellbeing offerings and identify areas for improvement. To ensure that the framework is both evidence-based and adaptable to various institutional contexts, it will be reviewed by a panel of wellbeing experts who have a background in each of the four domains of the framework. Feedback from the expert panel will be used to refine framework domains, clarify definitions, and ensure adaptability across diverse institutional contexts.

### Ethics and dissemination

Human Research Ethics Approval was obtained from the NYU Langone Health Institutional Review Board (IRB ID: i25-00965). The content analysis results and qualitative themes extracted from key informant and focus group interviews will be made available to all study participants. They will also be disseminated in a peer-reviewed journal.

### Status and timeline

No stages of the study have been completed at present. Participant recruitment is expected to begin February 2026 or after. Phase II recruitment is anticipated to be completed by May 2026, and Phase III recruitment by May 2026. Data collection will be completed by August 2026, and results are expected by April 2027.

## Discussion and limitations

Through this multiphase methodology, we will fill a critical gap in the wellbeing literature: the structure, design, and availability of medical school wellbeing programs. By integrating insights from a diverse group of stakeholders, we will propose a framework that medical schools can use to refine their wellbeing programs as they see fit. We hope that future research will build on and modify this framework in keeping with the latest developments in medical school wellbeing. Medical education is a critical juncture for future generations of healthcare leaders, and it is in the best interest of medicine for institutions to continue to remain cognizant of their future physicians’ holistic wellbeing by implementing evidence-based wellbeing programs.

With each phase, this study is expected to produce novel evidence that will be valuable to future design of medical student wellbeing programs. The web-based content analysis of publicly available institutional wellbeing program webpages will yield information about the scope, structure, and accessibility of formal wellbeing initiatives across a variety of wellbeing domains. KIIs with medical school leaders and administrators will reveal common rationales behind program design and implementation strategies, as well as the major institutional priorities that shape wellbeing programming. FGDs with medical students will capture student perceptions of program effectiveness or barriers and recommendations for improvement, thereby ensuring that the standardized framework for evaluating wellbeing programs emerging from this study reflects user-end realities.

Several limitations to this study protocol exist. Because the web-scraping process relies on publicly availability information, there may be selection bias that limits the full representation of wellbeing programs at all schools. For example, the implementation of wellbeing programs may not be fully captured because the availability of publicly available information does not guarantee that featured programs are actually being implemented; conversely, a lack of wellbeing information about a school does not necessarily indicate that wellbeing objectives are not a priority for the institution. Additionally, inconsistent or outdated web content may limit the scope and accuracy of content analysis. In Phase II and III, purposive sampling of stakeholders could lead to selection bias; moreover, interviews may be subject to self-reporting or recall bias, as participants may selectively report information or have difficulty accurately recalling program components, potentially leading to incomplete accounts during KIIs and FGDs.

The developed framework could also have limited applicability. First, the study’s resource constraints limit the number of schools and stakeholders included, and schools with certain features may be left out of the analysis by chance. Second, schools may be at different stages in their approach to wellbeing. Third, other cross-institutional differences, particularly regional and cultural variations, could also affect framework applicability. While this study focuses on wellbeing programs at the medical school level, it is important to note that medical training extends well beyond this stage, often lasting between 7 to 15 years including residency and fellowship. Challenges to wellbeing that emerge during medical school may persist and remain unaddressed throughout training and into professional practice, underscoring the need for longitudinal and system-wide approaches to student wellbeing.
